# RNA-Seq analysis of resistant and susceptible potato varieties during the early stages of potato virus Y infection

**DOI:** 10.1186/s12864-015-1666-2

**Published:** 2015-06-20

**Authors:** Aymeric Goyer, Launa Hamlin, James M. Crosslin, Alex Buchanan, Jeff H. Chang

**Affiliations:** Department of Botany and Plant Pathology, Oregon State University, Corvallis, OR USA; Hermiston Agricultural Research and Extension Center, Hermiston, OR USA; Center for Genome Research and Biocomputing, Oregon State University, Corvallis, OR USA; USDA-ARS, Prosser, WA USA

**Keywords:** Potato virus Y, Potato, RNA-Seq, Leaf, Resistance

## Abstract

**Background:**

Potato virus Y (PVY) is one of the most important plant viruses affecting potato production. The interactions between potato and PVY are complex and the outcome of the interactions depends on the potato genotype, the PVY strain, and the environmental conditions. A potato cultivar can induce resistance to a specific PVY strain, yet be susceptible to another. How a single potato cultivar responds to PVY in both compatible and incompatible interactions is not clear.

**Results:**

In this study, we used RNA-sequencing (RNA-Seq) to investigate and compare the transcriptional changes in leaves of potato upon inoculation with PVY. We used two potato varieties: Premier Russet, which is resistant to the PVY strain O (PVY^O^) but susceptible to the strain NTN (PVY^NTN^), and Russet Burbank, which is susceptible to all PVY strains that have been tested. Leaves were inoculated with PVY^O^ or PVY^NTN^, and samples were collected 4 and 10 h post inoculation (hpi). A larger number of differentially expressed (DE) genes were found in the compatible reactions compared to the incompatible reaction. For all treatments, the majority of DE genes were down-regulated at 4 hpi and up-regulated at 10 hpi. Gene Ontology enrichment analysis showed enrichment of the biological process GO term “Photosynthesis, light harvesting” specifically in PVY^O^-inoculated Premier Russet leaves, while the GO term “nucleosome assembly” was largely overrepresented in PVY^NTN^-inoculated Premier Russet leaves and PVY^O^-inoculated Russet Burbank leaves but not in PVY^O^-inoculated Premier Russet leaves. Fewer genes were DE over 4-fold in the incompatible reaction compared to the compatible reactions. Amongst these, five genes were DE only in PVY^O^-inoculated Premier Russet leaves, and all five were down-regulated. These genes are predicted to encode for a putative ABC transporter, a MYC2 transcription factor, a VQ-motif containing protein, a non-specific lipid-transfer protein, and a xyloglucan endotransglucosylase-hydroxylase.

**Conclusions:**

Our results show that the incompatible and compatible reactions in Premier Russet shared more similarities, in particular during the initial response, than the compatible reactions in the two different hosts. Our results identify potential key processes and genes that determine the fate of the reaction, compatible or incompatible, between PVY and its host.

**Electronic supplementary material:**

The online version of this article (doi:10.1186/s12864-015-1666-2) contains supplementary material, which is available to authorized users.

## Background

Potato (*Solanum tuberosum* L.) is one of the most consumed staple food crops worldwide, with a total world production of over 367 million tons in 2013, following maize, rice, and wheat (FAOSTAT data). Potatoes are cultivated in over 100 countries, under all latitudes, and from sea level up to 4,700 m above sea level. Per capita consumption is the highest in Europe and North America, but it has been dramatically increasing in southern and eastern Asia, where almost half of the world’s potato supply is consumed, as well as in Africa and Latin America. Potato is therefore a fundamental element of food security for millions of people. Since 2005, developing countries produce more than half of the global potato production. With the projected increased demand for food production in the next decades, dramatic increases in potato production are needed.

Potato virus Y (PVY) is one of the most important plant viruses affecting potato production [1]. PVY is an aphid-borne virus of the genus *Potyvirus* in the family *Potyviridae*. Nine PVY strains are currently known, O, C, N, E, N-Wi, N:O, NTN, NA-N, and NE-11 [2], which differ at the biological, serological, and molecular levels. Foliar and tuber symptoms associated with PVY vary greatly depending on the virus strain and the potato cultivar, ranging from no symptoms, local lesions, and mild mosaic to crinkling, systemic necrosis and death [3–5]. The molecular interactions between the host and the PVY strain during the early stages of infection determine the fate of the virus life and host health. In compatible reactions, the host defence system cannot prevent virus replication and movement and is called susceptible. In incompatible reactions, the host is able to prevent replication and movement of the virus and is called resistant. Incompatible reactions involve resistance genes. There are two types of PVY resistance genes: *R* genes which confer extreme resistance to all PVY strains and are present in the wild relatives of potato *Solanum tuberosum* ssp. *andigena*, *Solanum stoloniferum*, and *Solanum chacoense*, and *N* genes which confer PVY strain-specific hypersensitive resistance (HR) and are common in commercial potato cultivars that produce strain-specific HR reactions against PVY [1, 5].

In the United States, the most dominant PVY strain is PVY^O^, although this strain has been progressively replaced by necrotic strains in recent years. Some North American potato varieties are resistant to PVY^O^. This is the case of Premier Russet which shows no systemic virus infection and no foliar symptoms on systemic leaves upon inoculation with PVY^O^ [6]. Yet Premier Russet is susceptible to necrotic strains of PVY such as PVY^NTN^. Premier Russet is therefore a good model to compare the molecular host-virus interactions in both compatible and incompatible reactions within one single host, and further understand how certain PVY strains and not others are able to by-pass the plant defence system of the host. Large scale transcriptome analyses have been used to further the understanding of plant-virus interactions. Baebler et al. (2009) [7] used microarrays analysis to compare changes in gene expression in the incompatible reaction between the variety Santé which carries the *R* gene from *Solanum stoloniferum*, and the necrotic strain PVY^NTN^, and the compatible reaction between PVY-susceptible Igor variety and PVY^NTN^. More recently, Baebler et al. (2014) [8] also used microarrays to analyze changes in gene expression in the incompatible reaction between the variety Rywal which carries the *Ny-1* gene and is resistant to various PVY strains (PVY^O^, PVY^N^, PVY^N-Wi^, PVY^NTN^) and the strain PVY^N-Wi^. However, there is currently no report about the transcriptome response on either the PVY^O^ strain or North American varieties.

The *N* gene which triggers HR to PVY^O^ in Premier Russet is not known, and no PVY-associated *N* gene has ever been identified. However, the *Ny*_*tbr*_ gene which triggers HR to PVY^O^ in *Solanum tuberosum* USW2230 was mapped to chromosome 4 [9]. Premier Russet likely contains the *Ny*_*tbr*_ gene as well. The recently sequenced *Solanum tuberosum* group Phureja genome [10] has enabled to identify and locate disease resistance genes within the potato genome. The majority of disease resistance genes cloned to date belong to the NB-LRR family. The encoded proteins contain a nucleotide binding site and leucine-rich repeat domain. Recent studies have identified 755 NB-LRR genes in the sequenced potato genome [11–13]. One of these genes may be the yet-to-be-cloned *Ny*_*tbr*_ gene.

The objectives of this study were (1) to compare the early molecular responses of Premier Russet to PVY infection in both compatible (PVY^NTN^) and incompatible (PVY^O^) interactions, (2) to compare compatible reaction in Premier Russet with compatible reaction in another host, in this case the PVY^O^-susceptible Russet Burbank variety, and (3) to analyze the expression of predicted disease resistance genes in PVY^O^-resistant Premier Russet and PVY^O^-susceptible Russet Burbank. For this, we analyzed changes in mRNA expression 4 and 10 h post inoculation (hpi) with PVY by RNA-Seq. This next generation sequencing technology takes advantage of the recent sequencing of the potato genome [10] and was recently shown to be a valuable method for transcriptome dynamics analysis in tetraploid potato [14–17]. Our results show major differences in the gene expression response in Premier Russet vs. Russet Burbank upon PVY^O^ inoculation, while the early response of Premier Russet to either PVY strains was more similar. Our data also identified a small set of genes which likely play important roles in the establishment of the HR response in Premier Russet.

## Results

### Inoculation of potato plants with PVY and evaluation of the virus spread

Virus-free *in vitro* potato plantlets of the varieties Premier Russet and Russet Burbank were transplanted to pots containing soil and grown in a randomized complete block design in a greenhouse for a month before PVY inoculation. A day before PVY inoculation, all plants were tested for PVY by ELISA and all plants were negative. Six plants per treatment (mock and PVY^O^ treatments for both varieties, and PVY^NTN^ treatment in the case of Premier Russet) per variety were then inoculated mechanically. Four weeks after PVY-inoculation, all plants were tested for the presence of PVY by ELISA (Table 1). The virus could not be detected in any of the Premier Russet plants that were inoculated with PVY^O^ (as was the case of mock inoculation), while the virus was detected in five out of six plants that were inoculated with PVY^NTN^, confirming that Premier Russet prevents systemic spread of the PVY^O^ strain but not the PVY^NTN^ strain. For Russet Burbank, the virus was detected in all plants that were inoculated with PVY^O^ while no virus was detected in mock-inoculated plants, showing that Russet Burbank is susceptible to PVY^O^ and cannot contain the virus to the inoculated tissues.

**Table 1:** Number of systemically PVY-infected plants 4-weeks post-inoculation as determined by ELISA, and nature of the interaction

	PVY^O^			PVY^NTN^			Mock	
	Inoculated	Infected	Interaction	Inoculated	Infected	Interaction	Inoculated	Infected
PR	6	0	Incompatible	6	5	Compatible	4	0
RB	6	6	Compatible	-	-	-	3	0

*PR* premier russet, *RB* russet burbank

### Treatments sampled, RNA sequencing and mapping, and identified transcripts

**Fig. 1:**
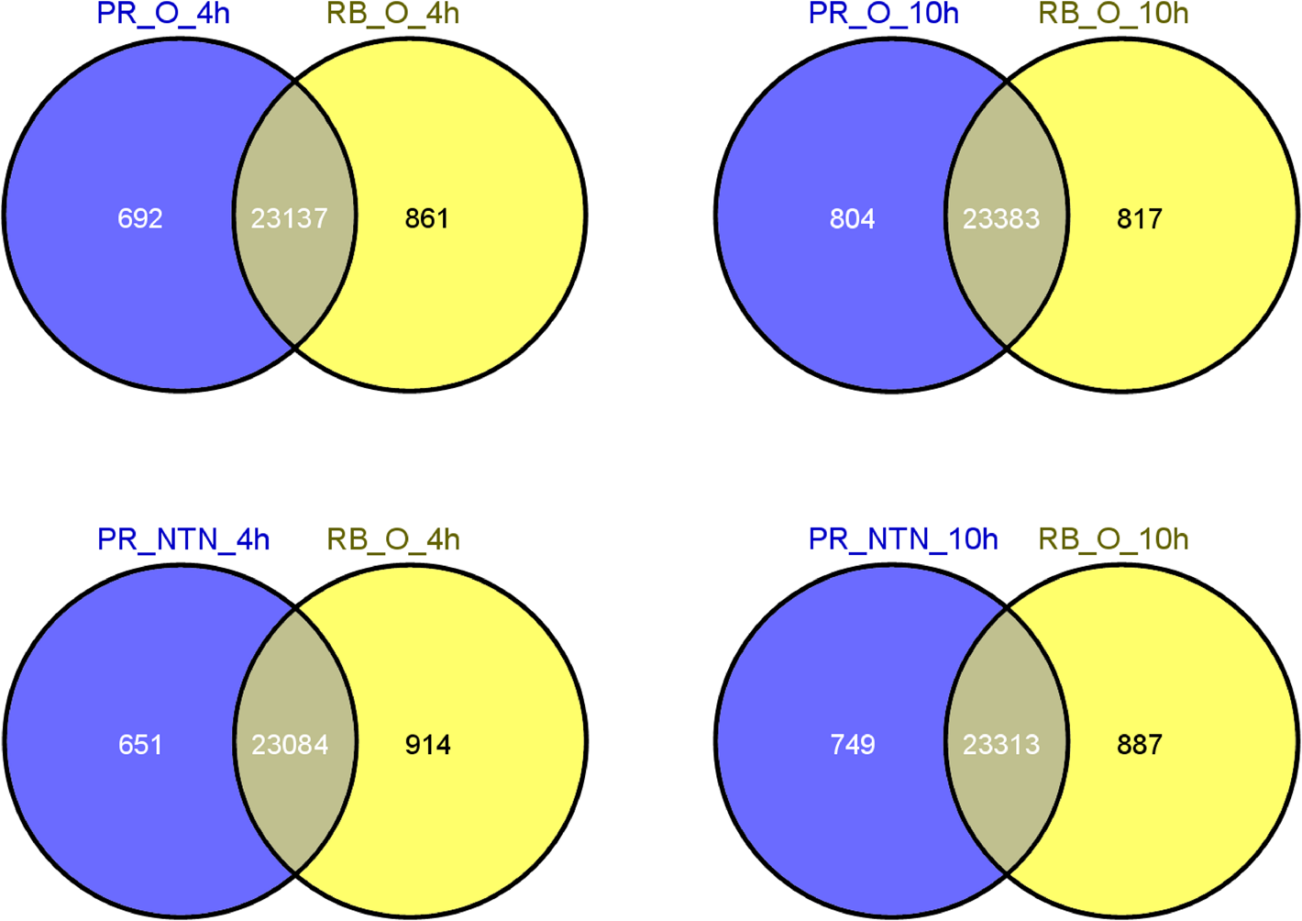
Venn diagrams showing the overlap of expressed genes between the two hosts at different time points (4 and 10 hpi) and inoculated with two different PVY strains, O and NTN

Leaves that were directly inoculated with PVY^O^, PVY^NTN^, or a mock solution were collected at 4 and 10 hpi. We chose 4 and 10 hpi sampling timing because we were interested in the early response of the plant to PVY infection. A first sampling at 4 hpi seemed adequate to observe significant changes in gene expression, because differences at earlier sampling times, *e.g.*, 0.5 hpi, may not have been as obvious, as previously reported [7]. Leaves were also collected before (0 hpi) PVY inoculation. A total of 34 leaf samples were analyzed (see Additional file 1). Altogether, over 1,086 million reads were generated, with the number of RNA-Seq reads per library ranging from 29.0 to 36.6 million. Mapping to the potato DM genome [10], transcript assembly, and quantification were performed by using two analytical pipelines: TopHat and Cufflinks [18], or JEANS, a modified version of GENE-counter [19]. These two different pipelines use different short read aligners, Bowtie (Burrows-Wheeler Transformation) and CASHX 2.3 [19], respectively. The number of transcripts identified as expressed and the number of high-confidence expressed transcripts were similar but not identical between the two methods (see Additional file 1). Only high-confidence expressed transcripts found by both methods were kept for further analysis. A total of 25,485 high-confidence transcripts were found across all the samples (see Additional file 1). The complement of expressed genes was similar between the two hosts Premier Russet and Russet Burbank (Fig. 1). Amongst the genes which were expressed specifically in Premier Russet or Russet Burbank, no more than one gene was DE after PVY inoculation.

### Differentially expressed genes in response to PVY inoculation in Premier Russet and Russet Burbank

**Fig. 2:**
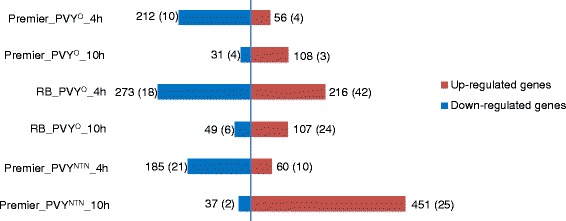
Number of up- and down-regulated genes in Premier Russet and Russet Burbank 4 and 10 h after inoculation with PVY^O^ or PVY^NTN^. Numbers in parenthesis indicate the number of genes whose |log_2_(FC)| was ≥ 2

Changes in transcript expression were analyzed with either the Cuffdiff program from Cufflinks [18] or NBPSeq [20]. These programs were chosen because they use different ways to model the negative binomial dispersion parameter [19, 21, 22]. Pairwise comparisons were made between PVY-inoculated vs. mock-inoculated samples at two time points, 4 and 10 hpi. A False Discovery Rate (FDR) cut-off of 5 % was used to select genes with significant differential expression. Only DE genes that were identified with both methods were kept for further analysis (Table 2). Overall, more genes were DE in compatible reaction than in incompatible reaction. More specifically, 645 and 407 genes were DE in PVY^O^-susceptible Russet Burbank and PVY^O^-resistant Premier Russet leaves, respectively, after inoculation with PVY^O^, and 733 genes were DE in PVY^NTN^-susceptible Premier Russet leaves inoculated with PVY^NTN^ (Table 2 and Additional file 2). For both varieties and with both PVY strains, more genes were down-regulated at 4 hpi than at 10 hpi while more genes were up-regulated at 10 hpi than at 4 hpi (Fig. 2). When comparing varieties and PVY treatments, the number of down-regulated genes was similar at each time point, while the number of up-regulated genes was very different. In particular, the number of up-regulated genes was much higher in the compatible reactions between PVY^O^ and PVY^O^-susceptible Russet Burbank at 4 hpi and between PVY^NTN^ and PVY^NTN^-susceptible Premier Russet at 10 hpi than in the incompatible reaction between PVY^O^ and PVY^O^-resistant Premier Russet (Fig. 2).

**Table 2:** Number of DE genes (p and q < 0.05) as determined by two different statistical programs (Cuffdiff and NBPSeq)

	Cuffdiff	NBPSeq	Number of DE genes identified by both methods	Number of DE genes with |log_2_(FC)| > 2
PR_PVY^O^_4h	506	321	268	14
PR_ PVY^O^ _10h	455	154	139	7
RB_ PVY^O^ _4h	1326	542	489	60
RB_ PVY^O^ _10h	819	182	156	30
PR_PVY^NTN^_4h	518	287	245	31
PR_ PVY^NTN^ _10h	791	593	488	27

**Fig. 3:**
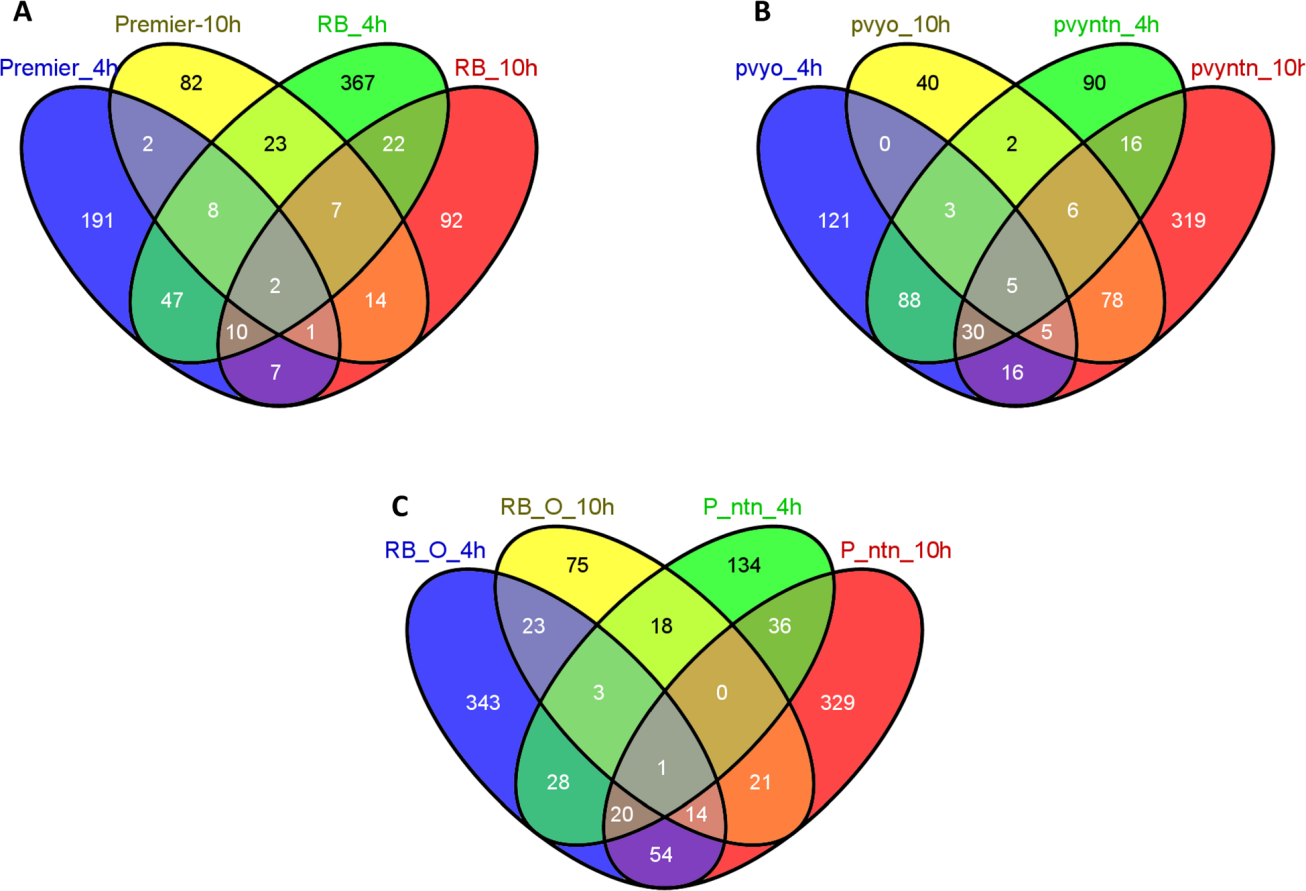
Venn diagrams showing the numbers of common and specific DE genes at different time points after PVY inoculation. **a**, Comparison between DE genes in Premier Russet and Russet Burbank (RB) 4 and 10 hpi with PVY^O^. **b**, Comparison between DE genes in Premier Russet 4 and 10 hpi with either PVY^O^ or PVY^NTN^. **c**, Comparison between DE genes in Russet Burbank and Premier Russet (P) 4 and 10 hpi with PVY^O^ or PVY^NTN^, respectively

The overlap of DE genes between treatments, time points, and hosts was analyzed and displayed in Venn diagrams (Fig. 3). Less than 25 and 18 % of genes that were DE at 4 or 10 hpi, respectively, in PVY^O^-resistant Premier Russet leaves inoculated with PVY^O^ were also DE in PVY^O^-susceptible Russet Burbank. Similarly, less than 22 and 8 % of genes DE at 4 or 10 hpi, respectively, in PVY^NTN^-susceptible Premier Russet leaves inoculated with PVY^NTN^ were also DE in PVY^O^-susceptible Russet Burbank leaves inoculated with PVY^O^. On the other hand, 47 and 67 % of genes DE at 4 or 10 hpi, respectively, in Premier Russet leaves inoculated with PVY^O^ were also DE in Premier Russet leaves inoculated with PVY^NTN^. These results show that there were more similarities in the response to PVY inoculation between compatible and incompatible reactions within one host than between compatible reactions in two different hosts.

In addition, the number of DE genes with a |log_2_(Fold Change (FC))| > 2 was much smaller in the incompatible reaction between Premier Russet and PVY^O^ than in the compatible reactions between Russet Burbank and PVY^O^ or Premier Russet and PVY^NTN^ (Table 2 and Additional file 3). In the incompatible reaction between Premier Russet and PVY^O^, 14 genes were DE with a |log_2_(FC)| > 2 at 4 hpi. Out of these 14 genes, one gene was up-regulated at 10 hpi as it was at 4 hpi while the expression of all 13 remaining genes was not significantly different at 10 hpi. At 10 hpi, 7 genes were DE with a |log_2_(FC)| > 2 but none of these genes were DE at 4 hpi. In the compatible reaction between Premier Russet and PVY^NTN^, 31 genes were DE with a |log_2_(FC)| > 2 at 4 hpi. Out of these 31 genes, 18 were not significantly DE at 10 hpi, 6 went from down-regulation at 4 hpi to up-regulation at 10 hpi, and 7 were consistently up-regulated at 4 and 10 hpi. At 10 hpi, 27 genes were DE with a |log_2_(FC)| > 2, 24 of these were not DE at 4 hpi, 2 were inversely regulated between 4 and 10 hpi, and 1 was consistently up-regulated at 4 and 10 hpi. In the compatible reaction between Russet Burbank and PVY^O^, 61 genes were DE with a |log_2_(FC)| > 2 at 4 hpi. Out of these 61 genes, 50 were not significantly DE at 10 hpi, 1 went from down-regulation at 4 hpi to up-regulation at 10 hpi, and 9 were consistently up- or down-regulated at 4 and 10 hpi. At 10 hpi, 30 genes were DE with a |log_2_(FC)| > 2, 20 of these were not DE at 4 hpi, 3 were inversely regulated between 4 and 10 hpi, and 7 were consistently up- or down-regulated at 4 and 10 hpi.

Out of the 14 DE genes with a |log_2_(FC)| > 2 in Premier Russet leaves inoculated with PVY^O^ at 4 hpi, 6 were similarly down-regulated in Premier Russet leaves inoculated with PVY^NTN^ at 4 hpi and 3 were similarly up-regulated in Russet Burbank leaves inoculated with PVY^O^ at 4 hpi (see Summary sheet in Additional file 3). Out of the 7 DE genes with a |log_2_(FC)| > 2 in Premier Russet leaves inoculated with PVY^O^ at 10 hpi, 3 and 2 genes were similarly up- or down- regulated, respectively, in Premier Russet leaves inoculated with PVY^NTN^ at 10 hpi, 2 genes were inversely either up- or down-regulated in Russet Burbank leaves inoculated with PVY^O^ at 4 hpi, and one gene was similarly upregulated in Russet Burbank leaves inoculated with PVY^O^ at 10 hpi (see Summary sheet in Additional file 3). Out of the 31 DE genes with a |log_2_(FC)| > 2 in Premier Russet leaves inoculated with PVY^NTN^ at 4 hpi, 11 were similarly up- or down-regulated and one was inversely regulated in Russet Burbank leaves inoculated with PVY^O^ at 4 hpi. Out of the 27 DE genes with a |log_2_(FC)| > 2 in Premier Russet leaves inoculated with PVY^NTN^ at 10 hpi, 9 were similarly up- or down-regulated and one was inversely regulated in Russet Burbank leaves inoculated with PVY^O^ at 10 hpi.

These results show that PVY^O^-resistant Premier Russet responds to PVY^O^ inoculation by changing the expression of fewer genes than PVY-susceptible Russet Burbank, in particular at 4 hpi, and that the great majority of these genes are DE in a variety-specific manner. These also show that Premier Russet responds to inoculation with both PVY^O^ and PVY^NTN^ strains by changing the expression of a large proportion of common genes between the two treatments, especially at 4 hpi, but the response becomes much more specific at 10 hpi in Premier Russet inoculated with PVY^NTN^. Among the DE genes with a |log_2_(FC)| > 2, only 5 were specific to Premier Russet inoculated with PVY^O^ (3 at 4 hpi and 2 at 10 hpi). These genes may play essential functions in the development of resistance to PVY^O^ in Premier Russet.

### Gene ontology enrichment analysis

**Fig. 4:**
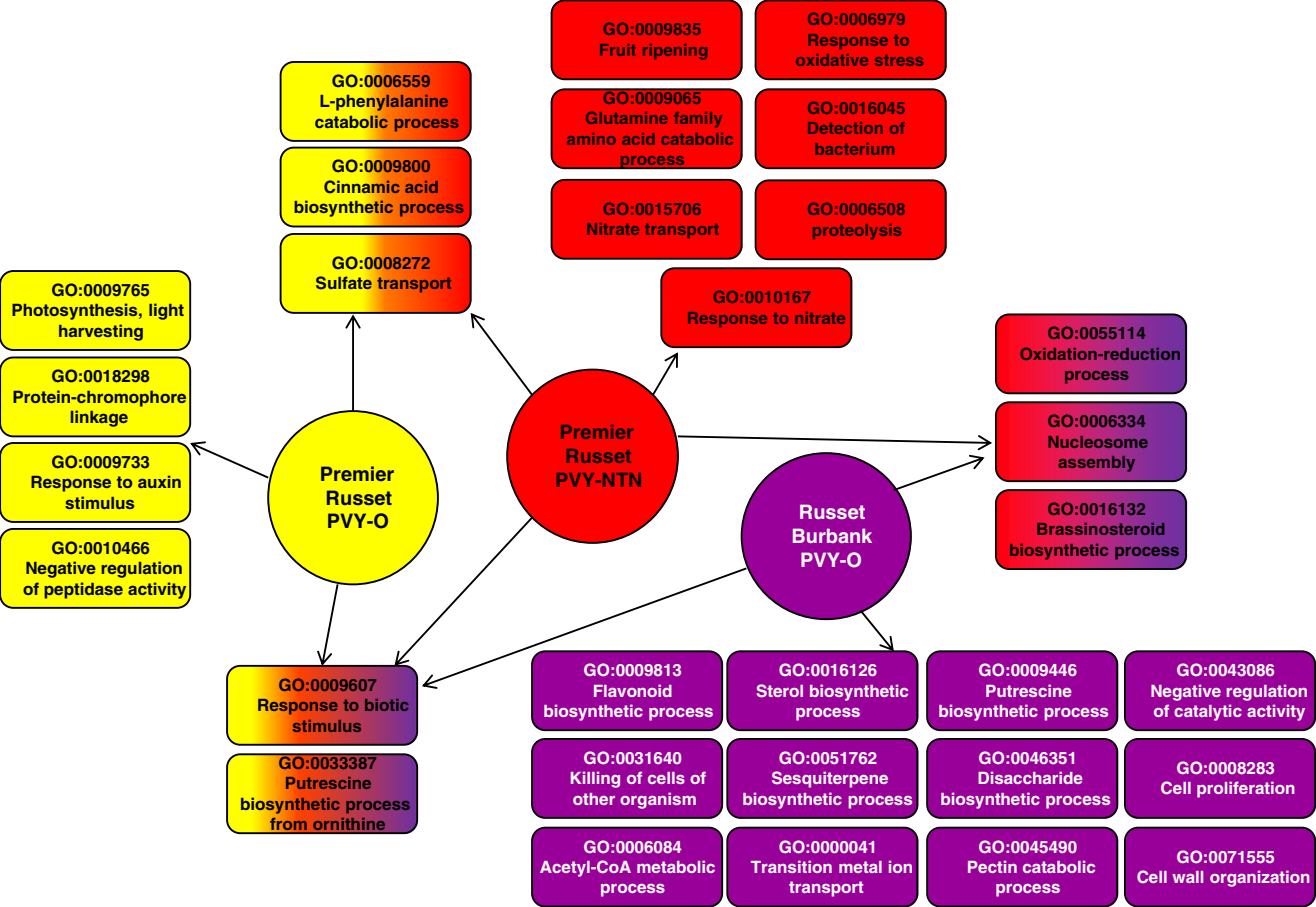
Gene Ontology terms (biological processes) that were enriched in incompatible and compatible reactions. Host and PVY strain are indicated in circles. GO terms are indicated in rectangles and are color-coded, *e.g.*, GO terms enriched only in the reaction between Premier Russet and PVY-O are indicated in yellow while GO terms enriched in reactions between Premier Russet and both PVY-O and PVY-NTN are in yellow and red

In order to find out in which functional categories DE genes belong to, we performed GO enrichment analysis using Blast2GO [23]. Out of the 25,485 high confidence expressed transcripts, 16,647 corresponding proteins were associated with at least one GO term. GO enrichment analysis of DE genes was performed for each treatment by using the corresponding high-confidence transcripts-encoded protein sequences as reference. For instance, for DE genes in Premier Russet at 4 hpi with PVY^O^, 23,829 proteins sequences corresponding to high-confidence transcripts expressed in Premier Russet at 4 hpi with PVY^O^ or mock were used as reference. Based on DE genes, a total of 69 GO terms were enriched across all samples in biological processes, molecular function, and cellular components (see Additional file 4), and at least 2 genes were associated with each GO term (see Additional file 4). Eight GO terms were enriched only in the incompatible reaction between Premier Russet and PVY^O^, 48 GO terms were specifically enriched in the compatible reactions between Premier Russet and PVY^NTN^ or Russet Burbank and PVY^O^ amongst which 11 were enriched in both compatible reactions. The biological process GO term “Photosynthesis, light harvesting” was the most significantly and specifically overrepresented term in Premier Russet leaves inoculated with PVY^O^ at 4 hpi (see Additional file 4 and Fig. 4). The enrichment in this biological process was reflected by enrichment in the molecular function GO term “chlorophyll binding” and the cellular component GO term “photosystem I” and “photosystem II” in PVY^O^-inoculated Premier Russet (see Additional file 4). Also unique to Premier Russet leaves inoculated with PVY^O^ at 4 hpi were the biological process GO terms “protein chromophore linkage”, “response to auxin stimulus”, “negative regulation of peptidase activity” (Fig. 4), and the molecular function GO term “N-acetyltransferase activity” (see Additional file 4). At 10 hpi in Premier Russet leaves inoculated with PVY^O^, only four GO terms (“putrescine biosynthetic process from ornithine”, “transferase activity, transferring hexosyl groups”, “oxidoreductase activity, acting on paired donors, with incorporation or reduction of molecular oxygen”, and “ornithine decarboxylase activator activity”) were overrepresented (see Additional file 4). These GO terms were also overrepresented in the compatible reactions between Premier Russet and PVY^NTN^ at 10 hpi and between Russet Burbank and PVY^O^ at either 4 or 10 hpi (see Additional file 4). GO terms in biological processes which were enriched in both compatible reactions and not in the incompatible reaction were “oxidation-reduction process”, “brassinosteroid biosynthetic process”, and “nucleosome assembly” (Fig. 4).

### Disease resistance genes

A list of 761 disease resistance genes was retrieved by combining data from the literature and keyword search in the potato genome annotation (see Additional file 5). This list includes the 456 NB-LRR genes previously reported [11, 12] plus 305 additional genes with disease resistance-related annotation. Expression of these genes was compared between PVY^O^-resistant Premier Russet and PVY^O^-susceptible Russet Burbank before (0 hpi) and after (4 and 10 hpi) PVY inoculation (Tables 3 and 4). A total of 25 genes had higher read counts in Premier Russet than in Russet Burbank, and 30 genes had lower read counts in Premier Russet than in Russet Burbank (Table 3). These genes were located across all chromosomes. Six genes which were more expressed in Premier Russet formed a cluster on chromosome 4 where the *Ny*_*tbr*_ gene was mapped. The expression of all genes but two, PGSC0003DMG400029415 and PGSC0003DMG400029586, did not change at 4 or 10 hpi. Only 7 of the disease resistance-related genes, 5 in Premier Russet and 2 in Russet Burbank, were DE after PVY inoculation, including the two genes previously mentioned (Table 4).

**Table 3:** Disease resistance annotated-genes which were DE (*p* < 0.05) between the two hosts before PVY inoculation

Gene ID	Pseudo counts PR	Pseudo counts RB	Ratio Pseudo counts PR over RB	Change of expression after PVY inoculation in PR	Change of expression after PVY inoculation in RB	Chr.
*PGSC0003DMG400006296*	68	0	Inf.	n.s.	n.s.	1
*PGSC0003DMG400006297*	24	10	2.4	n.s.	n.s.	1
*PGSC0003DMG400013094*	42	21	2.0	n.s.	n.s.	2
*PGSC0003DMG400024337*	8	0	Inf.	n.s.	n.s.	3
PGSC0003DMG400029452	116	42	2.8	n.s.	n.s.	4
PGSC0003DMG400029456	48	21	2.3	n.s.	n.s.	4
PGSC0003DMG400029457	68	25	2.7	n.s.	n.s.	4
PGSC0003DMG400029505	1018	281	3.6	n.s.	n.s.	4
PGSC0003DMG400029506	55	17	3.2	n.s.	n.s.	4
*PGSC0003DMG400029460*	43	13	3.3	n.s.	n.s.	4
PGSC0003DMG400013486	194	54	3.6	n.s.	n.s.	5
PGSC0003DMG400013490	1681	489	3.4	n.s.	n.s.	5
*PGSC0003DMG400025615*	16	0	Inf.	n.s.	n.s.	5
PGSC0003DMG400038713	15	0	Inf.	n.s.	n.s.	5
*PGSC0003DMG400033131*	238	127	1.9	n.s.	n.s.	6
*PGSC0003DMG400024206*	401	128	3.1	n.s.	n.s.	7
PGSC0003DMG400011907	16	0	Inf.	n.s.	n.s.	9
*PGSC0003DMG400011906*	20	0	Inf.	n.s.	n.s.	9
PGSC0003DMG400009272	9	5	1.8	n.s.	n.s.	11
PGSC0003DMG400027797	631	111	5.7	n.s.	n.s.	11
PGSC0003DMG401004578	65	17	3.8	n.s.	n.s.	12
PGSC0003DMG400029415	348	173	2.0	−0.79 (4 hpi O)	n.s.	12
				−1.06 (4 hpi NTN)		
PGSC0003DMG400007870	105	12	8.8	n.s.	n.s.	12
PGSC0003DMG400007872	162	24	6.8	n.s.	n.s.	12
*PGSC0003DMG400034825*	16	0	Inf.	n.s.	n.s.	12
PGSC0003DMG400030045	5	11	0.5	n.s.	n.s.	4
PGSC0003DMG400002217	141	383	0.4	n.s.	n.s.	4
*PGSC0003DMG401011522*	20	70	0.3	n.s.	n.s.	4
PGSC0003DMG400011527	35	89	0.4	n.s.	n.s.	4
*PGSC0003DMG401015877*	126	397	0.3	n.s.	n.s.	4
PGSC0003DMG400002961	32	81	0.4	n.s.	n.s.	4
PGSC0003DMG400003380	155	679	0.2	n.s.	n.s.	5
PGSC0003DMG400025547	14	32	0.4	n.s.	n.s.	5
PGSC0003DMG400005173	0	22	Inf.	n.s.	n.s.	6
PGSC0003DMG400033154	8	19	0.4	n.s.	n.s.	6
PGSC0003DMG400024203	0	14	Inf.	n.s.	n.s.	7
*PGSC0003DMG400029586*	0	51	Inf.	n.s.	+1.25 (4 hpi)	8
*PGSC0003DMG400029590*	32	119	0.3	n.s.	n.s.	8
*PGSC0003DMG400042937*	0	13	Inf.	n.s.	n.s.	8
PGSC0003DMG400002278	0	9	Inf.	n.s.	n.s.	8
PGSC0003DMG400002279	14	34	0.4	n.s.	n.s.	8
PGSC0003DMG400016599	13	136	0.1	n.s.	n.s.	9
PGSC0003DMG400016628	6	29	0.2	n.s.	n.s.	9
PGSC0003DMG401008349	40	137	0.3	n.s.	n.s.	10
PGSC0003DMG400011426	41	247	0.2	n.s.	n.s.	10
PGSC0003DMG403008349	85	264	0.3	n.s.	n.s.	10
PGSC0003DMG400019669	125	323	0.4	n.s.	n.s.	11
PGSC0003DMG400027410	0	78	Inf.	n.s.	n.s.	11
PGSC0003DMG400030239	8	25	0.3	n.s.	n.s.	11
PGSC0003DMG400030240	0	7	Inf.	n.s.	n.s.	11
*PGSC0003DMG400004295*	70	171	0.4	n.s.	n.s.	12
*PGSC0003DMG400024273*	17	104	0.2	n.s.	n.s.	12
PGSC0003DMG401029345	265	589	0.4	n.s.	n.s.	12
PGSC0003DMG400045101	0	17	Inf.	n.s.	n.s.	12
PGSC0003DMG400047046	26	85	0.3	n.s.	n.s.	12

Pseudo counts were determined with NBP-Seq. Genes which were identified as pseudogenes by Lozano et al. [11] are italicized

**Table 4:** Disease resistance annotated-genes which were DE (*p* < 0.05) after PVY inoculation

Gene ID	PR_PVY^O^		PR_PVY^NTN^		RB_PVY^O^		Chr.
	4 hpi	10 hpi	4 hpi	10 hpi	4 hpi	10 hpi	
PGSC0003DMG400029314	−1.10	n.s.	−1.42	n.s.	n.s.	n.s.	12
PGSC0003DMG400029415	−0.79	n.s.	−1.06	n.s.	n.s.	n.s.	12
PGSC0003DMG400024661	−1.00	n.s.	−1.08	n.s.	n.s.	n.s.	3
PGSC0003DMG400005542	−0.75	n.s.	n.s.	n.s.	n.s.	n.s.	12
PGSC0003DMG400008296	n.s.	n.s.	n.s.	+1.84	n.s.	n.s.	2
*PGSC0003DMG400029586*	n.s.	n.s.	n.s.	n.s.	+1.25	n.s.	8
PGSC0003DMG400044242	n.s.	n.s.	n.s.	n.s.	n.s.	+1.68	7

Data are |log_2_(FC)|. Genes which were identified as pseudogenes by Lozano et al. [11] are italicized

### Validation of differentially expressed genes by qRT-PCR

A subset of four genes which were DE in response to PVY was selected for quantitative real-time PCR (qRT-PCR) analyses (see Additional file 6). Twenty-three of 24 qRT-PCR analyses showed trends of expression, up- or down-regulation, similar to those found by RNA-Seq. The sample for which the trend could not be confirmed (PGSC0003DMG400024770 in Russet Burbank 10 hpi with PVY^O^) had low (|log_2_(FC)| ≤ 0.5), non-significant change of expression as determined by either RNA-Seq or qRT-PCR. Significance (p < 0.05 for qRT-PCR and q < 0.05 for RNA-Seq) was confirmed for 16 of the 24 analyses.

## Discussion

This study is, to our knowledge, the first large scale transcriptome RNA-Seq analysis of the response to the PVY^O^ strain in North American potato varieties and the first report on the molecular response of one single host to two different PVY strains. Our results provide new insights into the compatible and incompatible response of potato to one of its most damaging pathogens.

Four biological process GO terms were enriched in the incompatible reaction between PVY^O^ and Premier Russet but not in either compatible reaction between PVY^NTN^ and Premier Russet or PVY^O^ and Russet Burbank, *i.e.* “photosynthesis, light harvesting”, “protein-chromophore linkage”, “response to auxin stimulus”, and “negative regulation of peptidase activity”. Changes or lack of changes in these biological processes upon PVY inoculation are therefore essential in determining the nature, compatible or incompatible, of the reaction between PVY and its host. The GO term “photosynthesis, light harvesting” was the most enriched GO term in the incompatible reaction between PVY^O^ and Premier Russet and includes all the genes which were associated with “protein-chromophore linkage”. Baebler et al. [7] observed up-regulation of numerous photosynthesis-related genes in the incompatible reaction between PVY^NTN^ and the resistant variety Santé which carries *Ry*_*sto*_ gene as well as in the compatible reaction between PVY^NTN^ and the sensitive variety Igor at 0.5 hpi. Our results and theirs show that photosynthesis-related genes are important in incompatible reactions whether the host carries *R* or *N* gene. It was suggested that photosynthesis-related genes are up-regulated in response to elevated energy demand for the first response to stress [7]. That there was no enrichment in photosynthesis-related genes in the compatible reaction between PVY^NTN^ and Premier Russet while numerous photosynthesis-related genes were up-regulated in the compatible reaction between PVY^NTN^ and Igor may be due to the specific response of the host. Another possible explanation is timing because both our study and Baebler’s show down-regulation of photosynthesis-related genes at 10 and 12 hpi, respectively. Up-regulation of photosynthesis-related genes may have occurred in Premier Russet in response to PVY^NTN^ inoculation, but this may have happened earlier than 4 hpi.

There were 11 genes associated with the GO term “response to auxin stimulus” in Premier Russet inoculated with PVY^O^, but only 4 were actually DE in the incompatible reaction only. All four were DE at 4 hpi but not at 10 hpi. Two of these genes, PGSC0003DMG400002163 and PGSC0003DMG400002174, are glutathione-S-transferases and both were down-regulated. Some glutathione-S-transferases were shown to play a role in disease development in *Nicotiana benthamiana* following infection by *Colletotrichum destructivum* and *C. orbiculare* [24]. Glutathione S-transferases are SA-responsive genes. They belong to the immediate-early genes responsive to SA [25]. A third gene, PGSC0003DMG400005327, which was up-regulated, is an auxin-responsive protein IAA16, and a fourth gene, PGSC0003DMG400026159, which was down-regulated, is annotated as a calcium-binding protein pbp1-like. CaM-binding proteins play a role as either activator or repressor of disease resistance via the SA signaling pathway [25]. Changes in expression of these genes, *i.e.*, glutathione S-transferase and CaM-binding protein, indicate a SA-signaling pathway as was described in Rywal cultivar carrying the *Ny-1* gene [8]. Hormonal signaling involving crosstalks between auxins, salicylic acid, jasmonic acid, and ethylene is known to be essential in the response to pathogens.

Four genes associated with the GO term “negative regulation of peptidase activity” were DE in Premier Russet inoculated with PVY^O^ at 4 hpi. Two of them were also DE in Premier Russet inoculated with PVY^NTN^ at 10 hpi. None of these genes were DE in Russet Burbank. The two genes which were DE in the incompatible reaction only, PGSC0003DMG400005921 and PGSC0003DMG400005950, are cystatins or cysteine proteinases inhibitors. Both were up-regulated. The replication mechanism of potyviruses involves the activity of cysteine proteinases [26]. The cysteine proteinase domain is responsible for cleavage of the viral polyprotein at the HC-Pro/P3 junction. It is located in the C-proximal part of HC-Pro [27]. Cystatins can inhibit the replication mechanism of these viruses and have been used to engineer resistance against potyviruses in transgenic tobacco plants [26].

Our results also show that only five genes were DE with a |log_2_(FC)| > 2 uniquely in the incompatible reaction between PVY^O^ and Premier Russet. These genes may be essential in the establishment of HR response to PVY^O^. The gene PGSC0003DMG400014879, which is located on chromosome 3, is a putative ABC transporter. This gene had the largest change in expression amongst differentially expressed genes in the incompatible reaction between Premier Russet and PVY^O^. Some ABC transporters are known to play a role in resistance to pathogens. The ABC transporter Lr34 provides resistance to multiple fungal pathogens in wheat [28]. ABC transporters are highly expressed in barley upon inoculation with barley yellow dwarf virus [29]. That the expression of the potato gene was strongly repressed 4 h after PVY inoculation compared to the mock inoculation and returned to steady state levels at 10 hpi suggests a different mechanism for this ABC transporter in the response to PVY inoculation. The gene PGSC0003DMG400012237, which is located on chromosome 8, belongs to the MYC2 transcription factor family. MYC2 is a basic helix-loop-helix domain-containing TF and is a negative regulator of several jasmonic acid-responsive pathogens defence genes [30]. MYC2 mutant plants were shown to have increased resistance to *Plectosphaerella cucumerina*, *Botrytis cinerea*, *Fusarium oxysporum* [31, 32], and *Pseudomonas syringae* [33, 34]. Repression of MYC2 at 4 hpi suggests a similar role in potato. The gene PGSC0003DMG400009434, which is located on chromosome 2, is a VQ motif-containing protein [35–38]. Several reports have shown that VQ motif-containing proteins interact with WRKY transcription factors to activate defence genes. In Arabidopsis, the VQ motif-containing proteins SIGMA FACTOR BINDING PROTEIN1 (SIB1) and SIB2 recognize the C-terminal WRKY domain and stimulate the DNA binding activity of WRKY33 [36]. *sib1* and *sib2* mutants have compromised resistance to *Botrytis cinerea* while *SIB1*-overexpressing plants have enhanced resistance. VQ motif-containing proteins were shown to be substrates of the mitogen-activated protein kinases (MAPKs) MPK3 and MPK6 and to interact with WRKY transcription factors to activate defence genes [37]. The authors proposed models where VQ proteins act as negative or positive regulator of WRKY transcription factors activity. In another study, plants which overexpressed VQ20 were more sensitive to *Botrytis cinerea* or *Pseudomonas syringae*. The authors suggested that VQ20 is a negative regulator in plant defence responses [38]. The down-regulation of PGSC0003DMG400009434 gene expression at 4 hpi suggests a similar role and mechanism in potato. The gene PGSC0003DMG400031236, which is located on chromosome 10, is a non-specific lipid-transfer protein (nsLTP) belonging to the pathogenesis-related PR-14 protein family. nsLTPs have antibiotic activity against bacterial and fungal pathogens [39]. Some LTPs of barley are localized in the outer, epidermal cell layer of the exposed surfaces of the plant, and appear to provide the plant with a defensive-protein shield. nsLTPs’ function in response to viruses is unclear since the involvement of nsLTPs in response to pathogens was described only in bacteria and fungus. Repression of PGSC0003DMG400031236 expression at 4 hpi is opposite of what would be expected if the encoded protein functioned as a defensive shield. The gene PGSC0003DMG400017298, which is located on chromosome 7, is homolog to the Arabidopsis xyloglucan endotransglucosylase-hydroxylase XTH9 [40]. Glucanases are enzymes regulating the size exclusion limit and permeability of plasmodesmata and play a role in biotic stress [41]. They are members of the PR-2 family. XTH9-homolog in *Brassica campestris*, *Bc*XTH1, is associated with cell expansion [42]. Arabidopsis plants overexpressing *Bc*XTH1 have a pronounced cell expansion phenotype. The expression of the XTH9 potato homolog was repressed at 4 hpi. Future investigation is warranted to characterize the exact function of these genes in the response of potato to PVY.

Amongst disease resistance-annotated genes, 55 were more expressed in either Premier Russet or Russet Burbank before inoculation with PVY (Table 3). These included five genes (and one pseudogene) which form a cluster in a distal region of chromosome 4 and were all more expressed in Premier Russet than in Russet Burbank. Although located on the same chromosome than the *Ny*_*tbr*_ resistance gene, the *Ny*_*tbr*_ gene was mapped between two markers, TG506 and TG208 [9], which are located in a more central region of chromosome 4. Therefore, this strongly rules out against any of these genes being the *Ny*_*tbr*_ gene. Only seven of the disease resistance-annotated genes were DE after PVY inoculation (Table 4), four in Premier Russet and three in Russet Burbank. All four genes which were DE in Premier Russet were down-regulated, and three of them were DE in both the compatible and incompatible reactions. All three genes which were DE in Russet Burbank were up-regulated. It is unclear at this point what role, if any, these genes play and how important they are in the response to PVY inoculation. However, none of these genes mapped to chromosome 4. Therefore, it seems unlikely that any of these genes is responsible for the resistance of Premier Russet to PVY^O^. Our analysis did not include the additional 331 NB-LRR sequences recently identified by Jupe et al. [13] which are absent from the original potato genome annotation. Amongst these NB-LRRs, 18 are located between TG506 and TG208 on chromosome 4. It would be interesting to analyze the expression of these genes in Premier Russet and Russet Burbank and upon PVY inoculation. In addition, commercial cultivars used in this study may contain *N* genes that are not present in the potato genome reference. These genes could be identified by *de novo* assembly and mapping of RNA-Seq reads generated in this study.

## Conclusions

In the present study, the response of two North American potato varieties, Premier Russet which is PVY^O^-resistant and Russet Burbank which is susceptible to all PVY strains, to two different PVY strains, the ordinary strain PVY^O^ and the necrotic strain PVY^NTN^, was analyzed at the transcriptome level by RNA-Seq. More similarities were found between the incompatible and compatible reactions within one host, Premier Russet, in the early response to PVY inoculation than between the two compatible reactions involving two different hosts. GO enrichment analysis revealed biological processes that are essential in the establishment of resistance to PVY, and showed how two different PVY strains trigger a different cascade of molecular changes. Further investigation is warranted to elucidate the specific functions of genes whose expression changed the most after PVY inoculation and/or that belong to GO terms enriched specifically in the incompatible reactions. These genes may be useful in breeding programs to develop PVY-resistant varieties.

## Methods

### Plant material

*In vitro* plantlets of the potato varieties Premier Russet and Russet Burbank were transplanted to 3-l pots containing Sunshine Mix1 supplemented with Osmocote on April 13, 2012 and were grown in a greenhouse under artificial light until 3 days before PVY inoculation. Greenhouse temperature was set at 27 °C. Plants were grown in a randomized complete block design until inoculation at which point plants were grouped per inoculation type (mock, PVY^O^, PVY^NTN^). Leaf samples were collected just before inoculation for PVY ELISA testing. All plants tested PVY-negative.

### PVY inoculation and sampling

The inoculum was prepared by grinding 0.2 g of PVY-infected tobacco leaves in 20 ml of cold 30 mM potassium phosphate buffer, pH 8.0. Six plants per treatment (treatment = mock or PVY inoculation) for each variety were inoculated. Three leaves per plant from the medium canopy level were marked with ties for rapid identification of leaves to inoculate and harvest. All leaflets per marked leaf were inoculated on the adaxial side. Leaflets were sprayed with carborundum and infected by rubbing the inoculum with pestle on the whole leaflet surface area. Four mechanically-inoculated leaflets, two from each side of the petiole, were collected from three plants (=three biological replicates which correspond to replicates described in Additional file 1) per treatment per variety at each time point, 4 and 10 hpi. The four harvested leaflets (each leaflet was about 0.3 to 0.5 g) per plant were pooled and frozen immediately in liquid nitrogen. All three biological replicated samples were used for RNA extraction (= three independent biological replicates (see Additional file 1)), except in the case of PVY^NTN^ where two samples were analyzed.

### RNA extraction

RNA was isolated using a phenol method [10]. Samples were treated with DNase (Ambion® DNA-*free*™ kit, Life Technologies). Quality of total RNAs was verified on an Agilent 2100 Bioanalyzer (Plant RNA Nano Chip, Agilent) and based on the rRNA ratio 25S/18S, RNA Integrity Number, and the absence of smear.

### RNA-Seq

A balanced block design was used for RNA-Seq analysis [43] (see Additional file 1). Samples were bar coded, pooled, processed together, and split for sequencing in two Illumina HiSeq2000 lanes (51-cycle v3 Single End). Illumina library preparation was done at the Center for Genome Research and Biocomputing at Oregon State University using TruSeq RNA. Illumina libraries were quantified by qPCR for optimal cluster density. Mapping of the RNA-Seq reads to the DM potato reference genome [10], transcript assembly, and determination of differences in expression levels were performed using TopHat and Cufflinks [18] or JEANS, a modified version of GENE-counter [19], in combination with NBPSeq [20]. With TopHat, a maximum of 20 multiple alignments to the reference for a given read (default option) and two mismatches per 50-bp reads were allowed (default option). High-confidence transcripts were obtained from identified transcripts (*i.e.*, transcripts with FPKM value in the case of cufflinks or pseudo-count in the case of GENE-counter > 0) by filtering for a FPKM 95 % confidence interval lower boundary greater than zero and FPKM value ≥ 0.001, or for pseudo-counts > 4. A FDR cut-off of 5 % was used to select genes with significant differential expression. Cross-replicate variability was evaluated by visualizing the squared coefficient of variation for each sample (see Additional file 7).

### Quantitative RT-PCR

RNAs (1 μg) were reverse-transcribed to cDNAs with M-MuLV Reverse Transcriptase (New England BioLabs). cDNAs were diluted twice in water and 1–4 μl of cDNAs were used as template in 25-μl PCR reactions containing the Brilliant II SYBR® Green QPCR Master Mix (Agilent Technologies) and 150 nM of forward and reverse primers (see Additional file 8). PCR reactions were performed on an Mx3005P instrument (Agilent Technologies). PCR conditions were: denaturation at 95 °C for 10 min, followed by 44 cycles at 95 °C for 30s, 58 °C for 30s, and 72 °C for 30s. A dissociation step (1 min at 95 °C, ramping down to 55 °C and up to 95 °C) was added at the end of the amplification cycles to check for primers specificity. The housekeeping gene ef1α (PGSC0003DMG400023270) was used as control for normalization of qPCR analysis [44]. Primers efficiencies were determined for each pair of primers using the protocol described in [45]. Relative gene expression was calculated by using the 2^-ΔΔCt^ method [45].

### Gene annotation, Gene Ontology enrichment, and Venn diagrams

Gene annotation was done with Blast2GO [23]. BLASTp was used to find sequence similarities with a cutoff of 1 × 10^−3^. GO annotation used an E-value hit filter of 1 × 10^−6^ and an annotation cutoff of 55. GO terms retrieved with InterPro were merged to the already existent GO terms. Statistical results for Blast, mapping, annotation, and InterPro annotation steps are in the Additional file 9. GO annotation is in the Additional file 10. GO enrichment was done with Blast2GO by using Fisher’s Exact Test with Multiple Testing Correction of FDR (Benjamini and Hochberg) at a cutoff of 0.05. Protein sequences corresponding to high-confidence transcripts of each specific treatment (*e.g.*, Premier Russet 4 hpi with PVY^O^) were used as reference. Venn Diagrams were done with VENNY [46].

### Availability of supporting data

Clean Illumina sequences were deposited at the NCBI Sequence Read Archive under the accessions SRP058212 and SRP058230.

## Additional files

Additional file 1:
**Number of RNA-Seq reads and expressed genes in 34 samples analyzed in this study.**
Additional file 2:
**Quantification of transcripts and differential expression.**
Additional file 3:
**Highly DE genes.** In the “Summary” sheet, genes that are up-regulated with a log_2_(FC) > 2 are highlighted in red; genes that are down-regulated with a log_2_(FC) < −2 are highlighted in light blue. DE genes that are only found in each specific treatment are in bold. In some cases, genes that had a |log_2_(FC)| > 2 with Cufflinks had a |log_2_(FC)| < 2 with NBP-Seq. In those cases, we kept genes that had a |log_2_(FC)| within 5 % of 2. Additional file 4:
**Gene Ontology enrichment.** In the summary sheet, numbers indicate the percentage of DE genes assigned to certain GO term (the first number corresponds to the test, and the number in parenthesis corresponds to the reference). Additional file 5:
**List of disease resistance-annotated genes.**
Additional file 6:
**Changes in expression of four selected genes as determined by qRT-PCR and comparison with RNA-Seq results.** Four genes were selected for qRT-PCR analysis. Asterisks indicate significance of fold changes in terms of FDR corrected q-values for RNA-seq data and p-values according to a student’s *t*-test for qRT-PCR data; * ≤0.05, ** ≤0.01, *** ≤0.005. Data are based on biological and technical triplicates. Additional file 7:
**Squares of coefficient of variation were determined for each treatment by using the R package cummeRbund.**
Additional file 8:
**List of oligonucleotide primers used for qRT-PCR experiments.**
Additional file 9:
**Blast, mapping, annotation, and InterPro statistics.**
Additional file 10:
**Gene Ontology annotation.**
