# Supplementary material for: RNA-Seq analysis of resistant and susceptible potato varieties during the early stages of potato virus Y infection
Source: BMC Genomics. 2015 Jun 20;16(1):472. doi: 10.1186/s12864-015-1666-2 (PMC4475319; doi:10.1186/s12864-015-1666-2)
Supplement: Additional file 6: — Changes in expression of four selected genes as determined by qRT-PCR and comparison with RNA-Seq results. Four genes were selected for qRT-PCR analysis. Asterisks indicate significance of fold changes in terms of FDR corrected q-values for RNA-seq data and p-values according to a student’s t-test for qRT-PCR data; * ≤0.05, ** ≤0.01, *** ≤0.005. Data are based on biological and technical triplicates. [file 12864_2015_1666_MOESM6_ESM.pptx]

## Slide 1
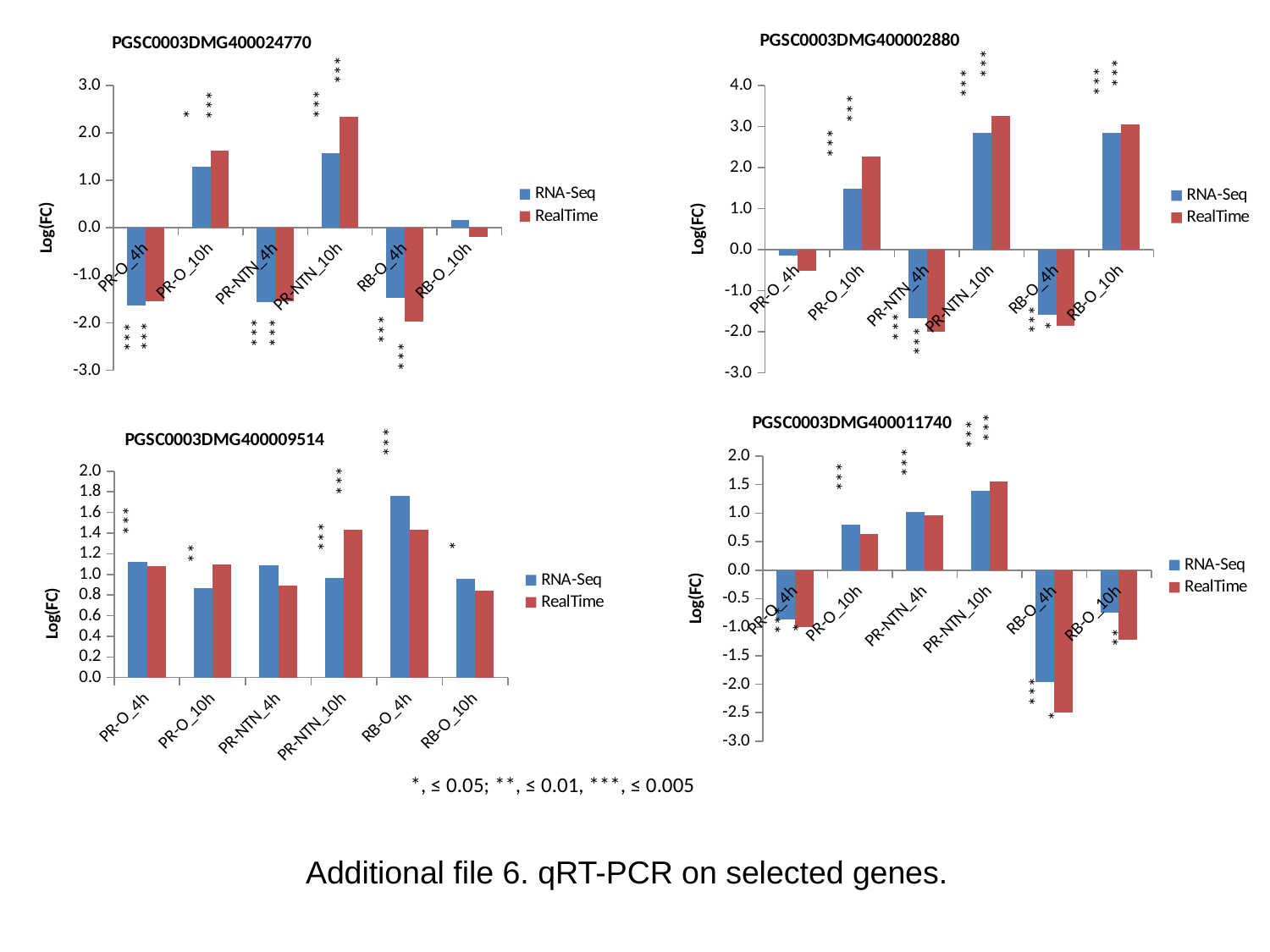

### Chart: PGSC0003DMG400024770
| Category | RNA-Seq | RealTime |
|---|---|---|
| PR-O_4h | -1.6373684861383395 | -1.5488888888888896 |
| PR-O_10h | 1.278078088839245 | 1.619999999999999 |
| PR-NTN_4h | -1.56406 | -1.54 |
| PR-NTN_10h | 1.57836 | 2.330000000000001 |
| RB-O_4h | -1.47977 | -1.981111111111112 |
| RB-O_10h | 0.16 | -0.1844444444444439 |
### Chart: PGSC0003DMG400002880
| Category | RNA-Seq | RealTime |
|---|---|---|
| PR-O_4h | -0.14 | -0.5133333333333329 |
| PR-O_10h | 1.4909008510763595 | 2.262222222222221 |
| PR-NTN_4h | -1.66587 | -1.99 |
| PR-NTN_10h | 2.83598 | 3.2511111111111104 |
| RB-O_4h | -1.58614 | -1.859999999999999 |
| RB-O_10h | 2.84238 | 3.0494444444444464 |***
***
***
***
***
***
***
***
*
***
***
*
***
***
***
***
***
***
***
***
### Chart: PGSC0003DMG400011740
| Category | RNA-Seq | RealTime |
|---|---|---|
| PR-O_4h | -0.8675992523844758 | -1.001 |
| PR-O_10h | 0.7913337598166317 | 0.6344444444444451 |
| PR-NTN_4h | 1.01607 | 0.96 |
| PR-NTN_10h | 1.38559 | 1.5611111111111122 |
| RB-O_4h | -1.95927 | -2.4955555555555557 |
| RB-O_10h | -0.75 | -1.2216666666666667 |***
### Chart: PGSC0003DMG400009514
| Category | RNA-Seq | RealTime |
|---|---|---|
| PR-O_4h | 1.1183355138104092 | 1.0766666666666644 |
| PR-O_10h | 0.866663593091923 | 1.0933333333333337 |
| PR-NTN_4h | 1.09 | 0.8944444444444439 |
| PR-NTN_10h | 0.961555 | 1.4288888888888913 |
| RB-O_4h | 1.76201 | 1.4355555555555553 |
| RB-O_10h | 0.961192 | 0.8388888888888896 |***
***
***
***
***
***
***
*
**
***
*
**
***
*
*, ≤ 0.05; **, ≤ 0.01, ***, ≤ 0.005
Additional file 6. qRT-PCR on selected genes.
