# Supplementary material for: RNA-Seq analysis of resistant and susceptible potato varieties during the early stages of potato virus Y infection
Source: BMC Genomics. 2015 Jun 20;16(1):472. doi: 10.1186/s12864-015-1666-2 (PMC4475319; doi:10.1186/s12864-015-1666-2)
Supplement: Additional file 7: — Squares of coefficient of variation were determined for each treatment by using the R package cummeRbund. [file 12864_2015_1666_MOESM7_ESM.pptx]

## Slide 1
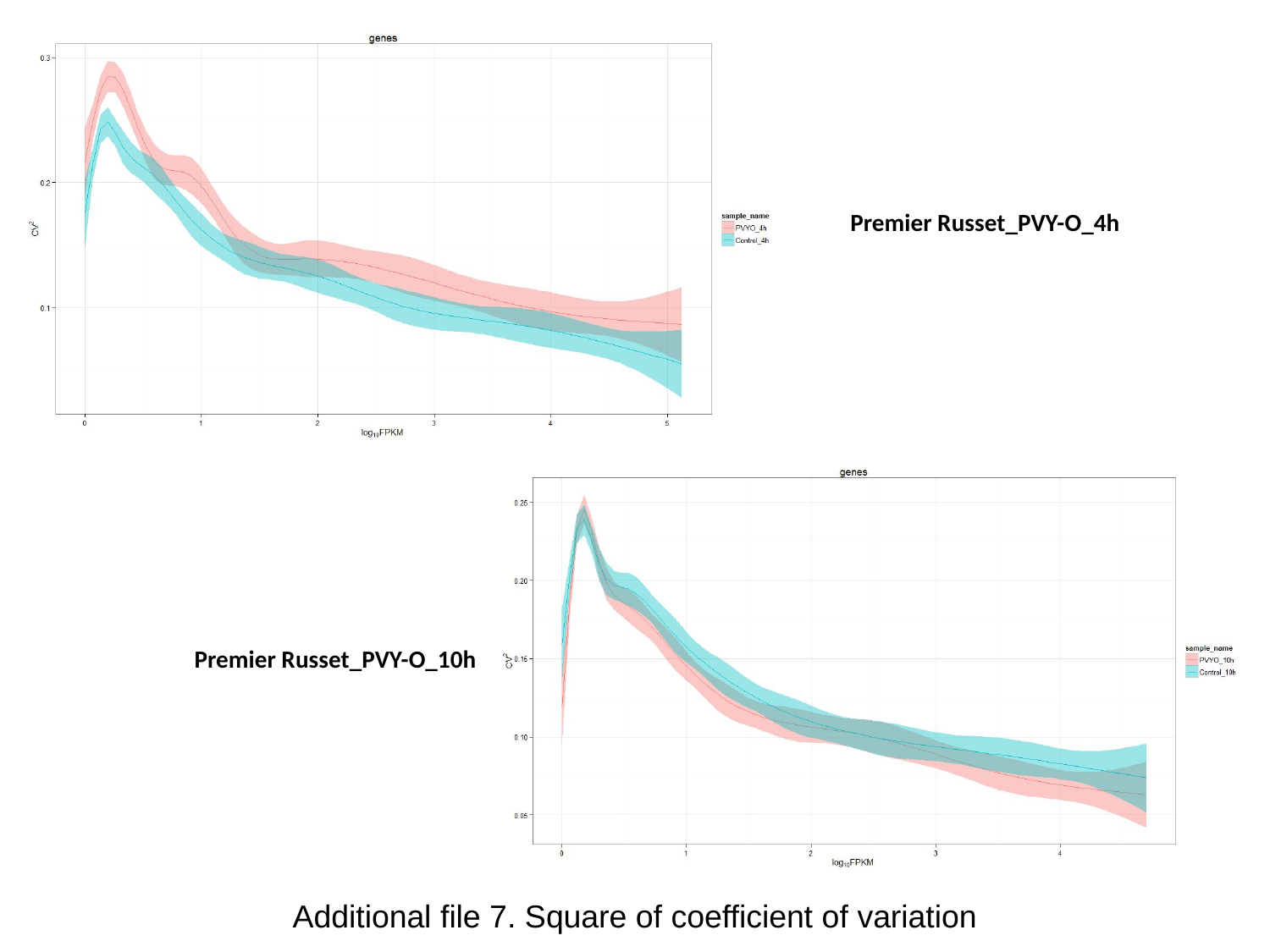

Premier Russet_PVY-O_4h
Premier Russet_PVY-O_10h
Additional file 7. Square of coefficient of variation

## Slide 2
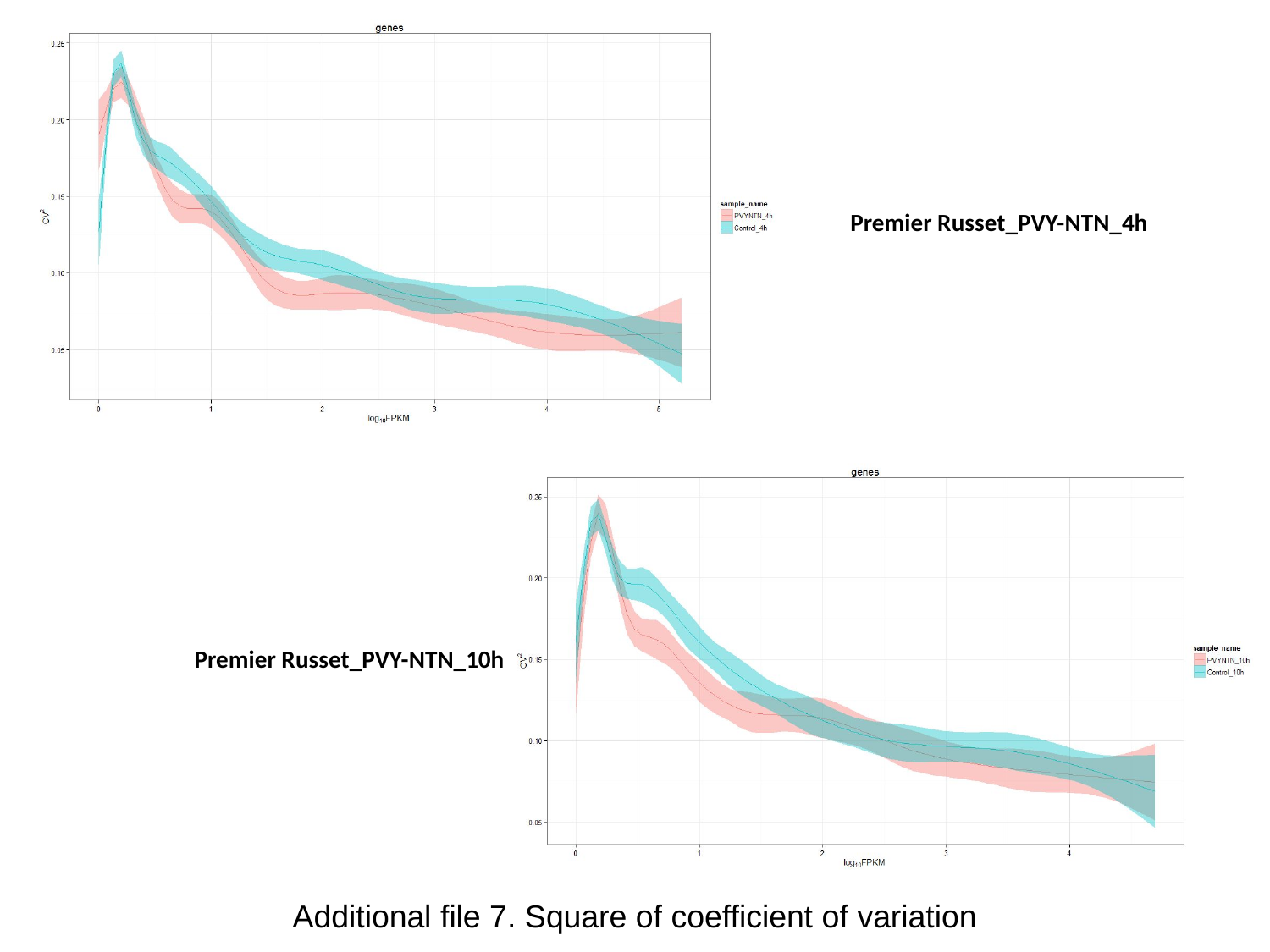

Premier Russet_PVY-NTN_4h
Premier Russet_PVY-NTN_10h
Additional file 7. Square of coefficient of variation

## Slide 3
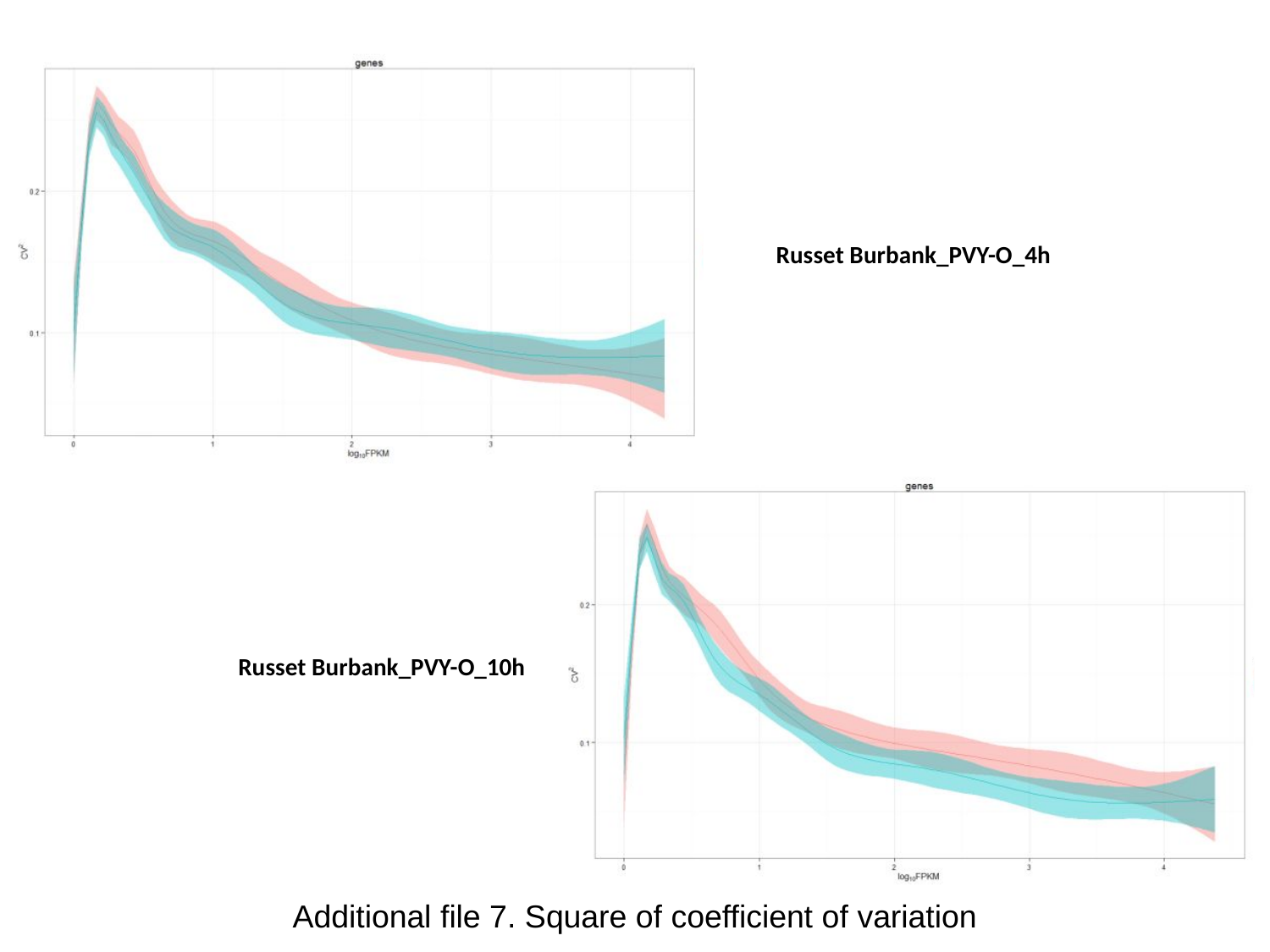

Russet Burbank_PVY-O_4h
Russet Burbank_PVY-O_10h
Additional file 7. Square of coefficient of variation

## Slide 4
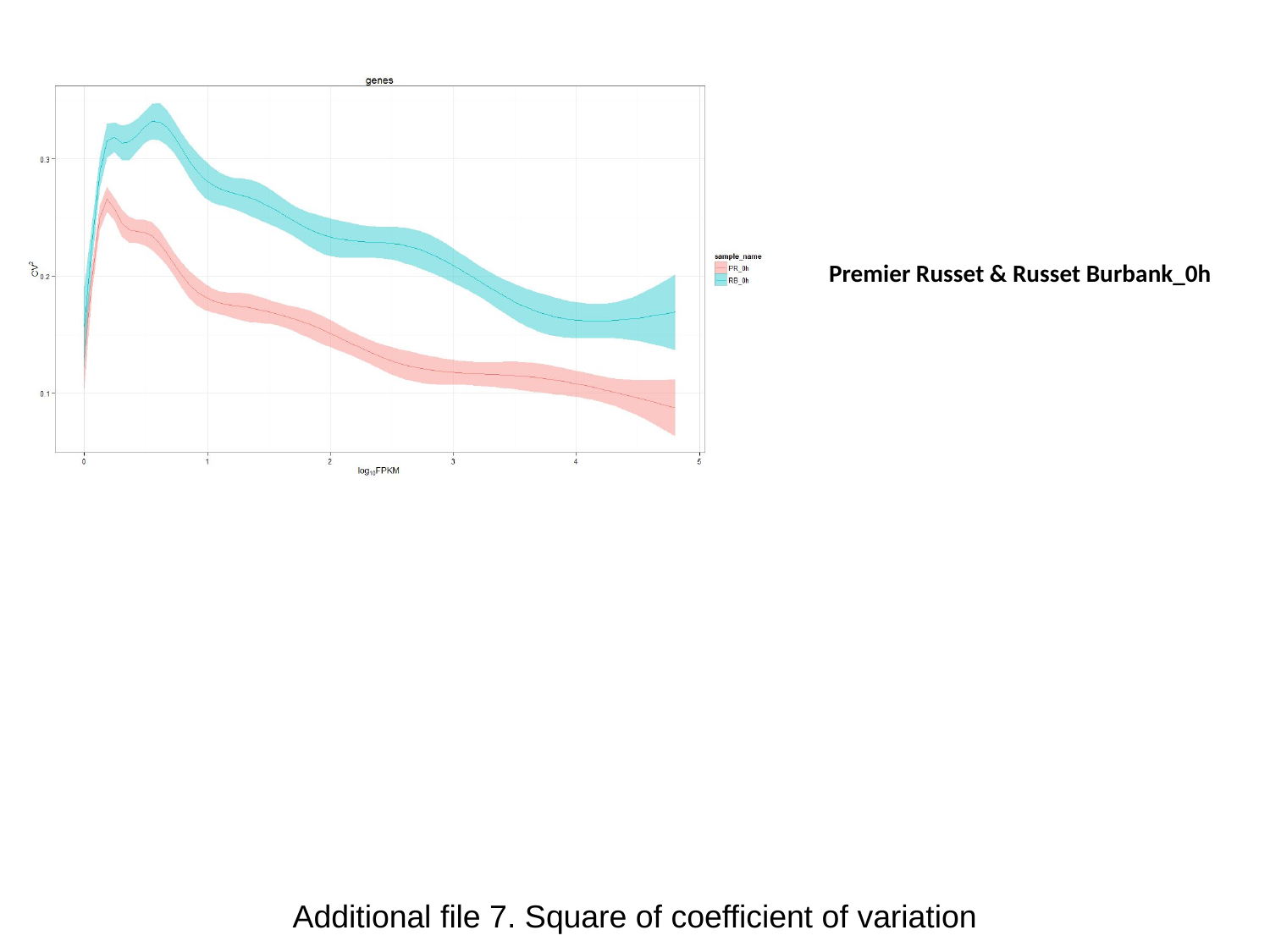

Premier Russet & Russet Burbank_0h
Additional file 7. Square of coefficient of variation
