# Supplementary material for: RNA-Seq analysis of resistant and susceptible potato varieties during the early stages of potato virus Y infection
Source: BMC Genomics. 2015 Jun 20;16(1):472. doi: 10.1186/s12864-015-1666-2 (PMC4475319; doi:10.1186/s12864-015-1666-2)
Supplement: Additional file 9: — Blast, mapping, annotation, and InterPro statistics. [file 12864_2015_1666_MOESM9_ESM.pptx]

## Slide 1
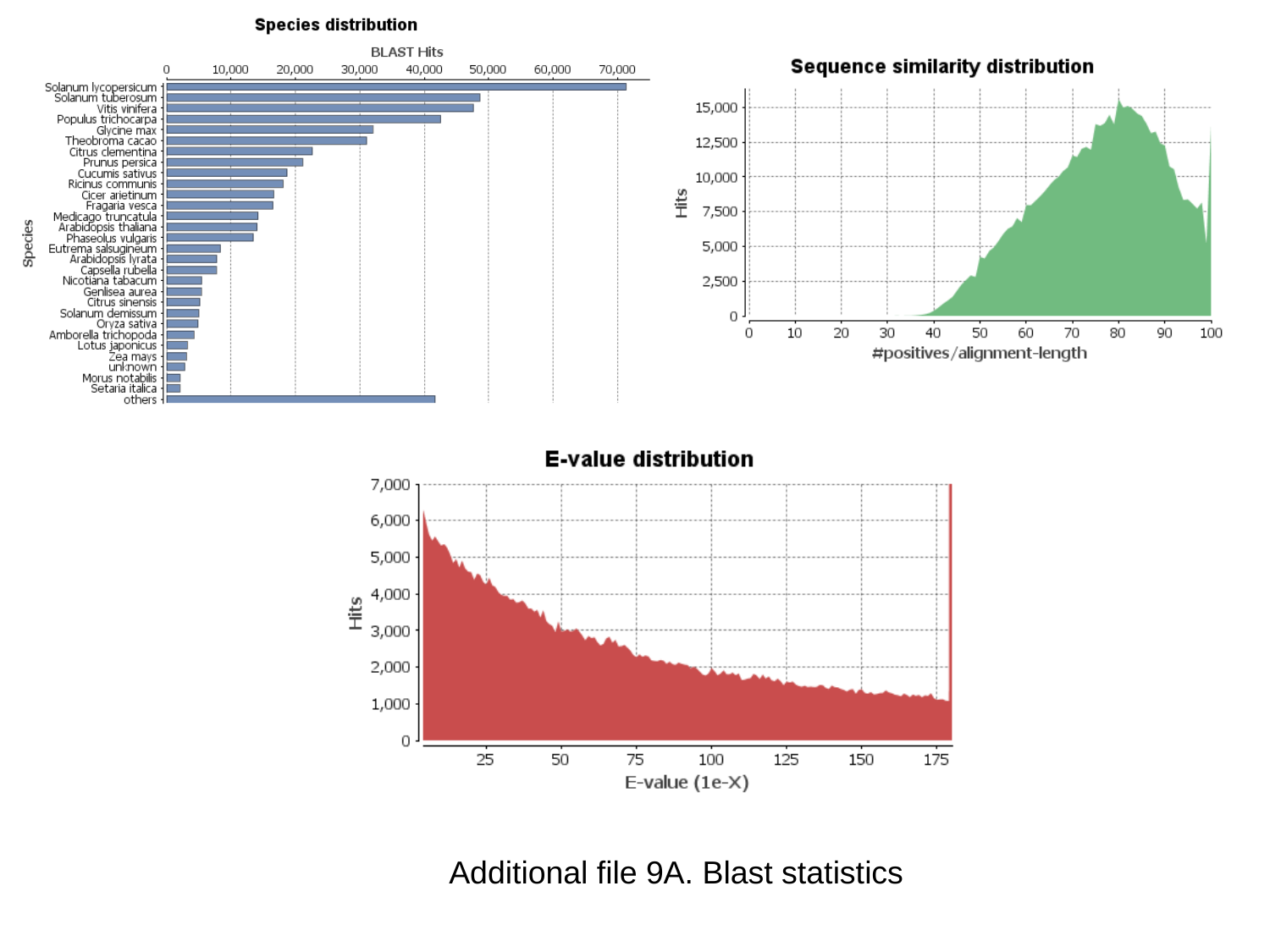

Additional file 9A. Blast statistics

## Slide 2
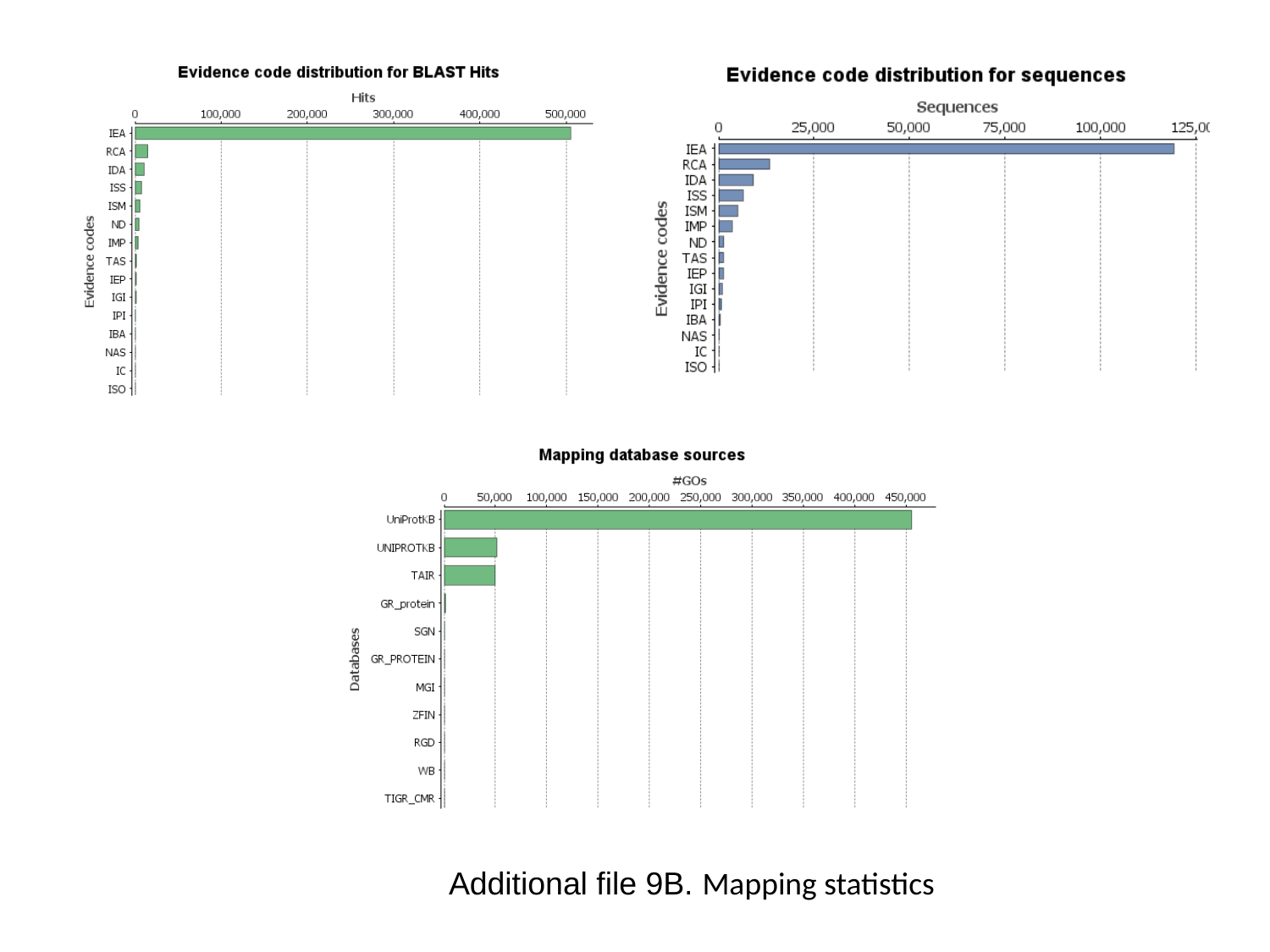

Additional file 9B. Mapping statistics

## Slide 3
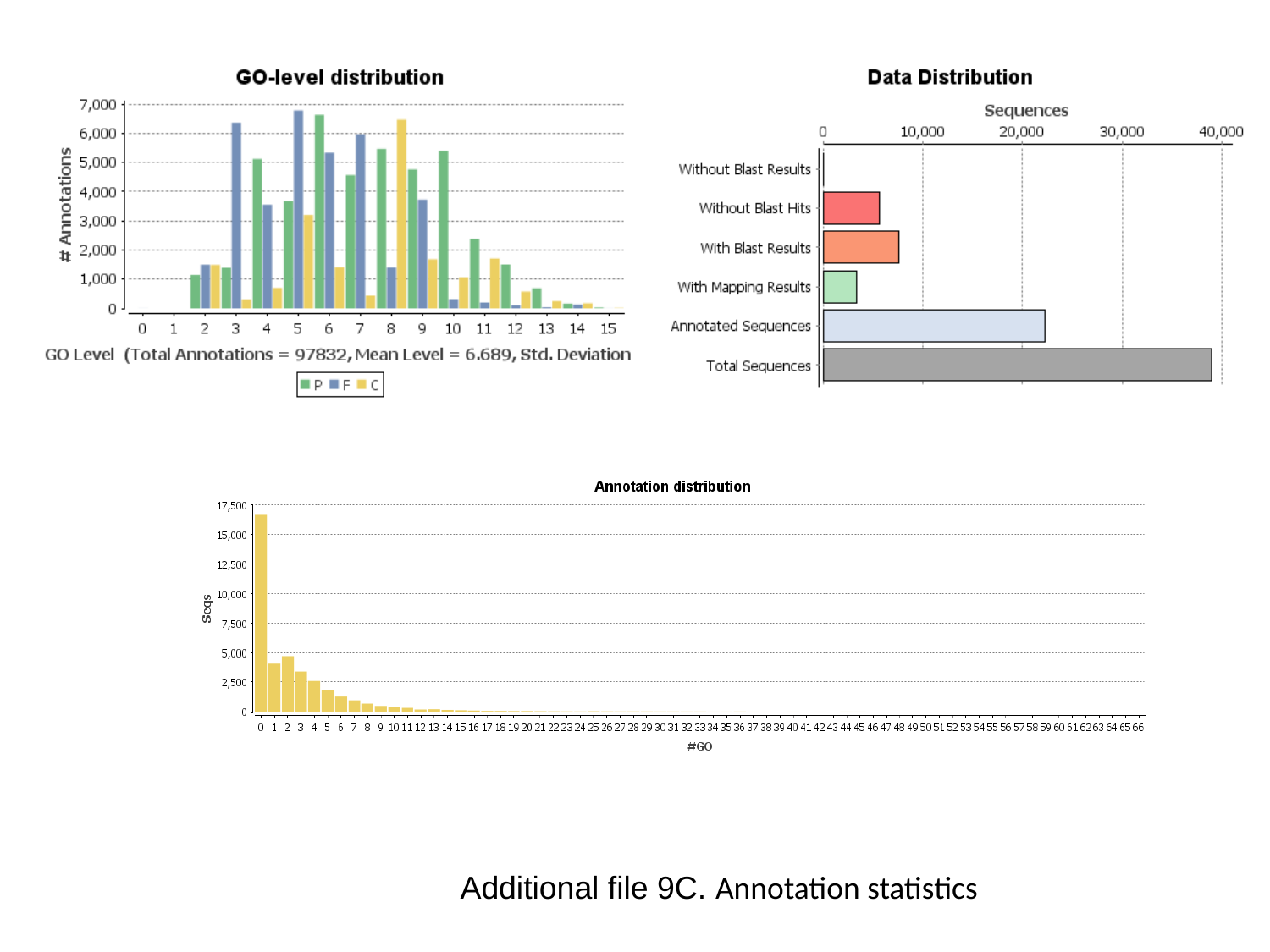

Additional file 9C. Annotation statistics

## Slide 4
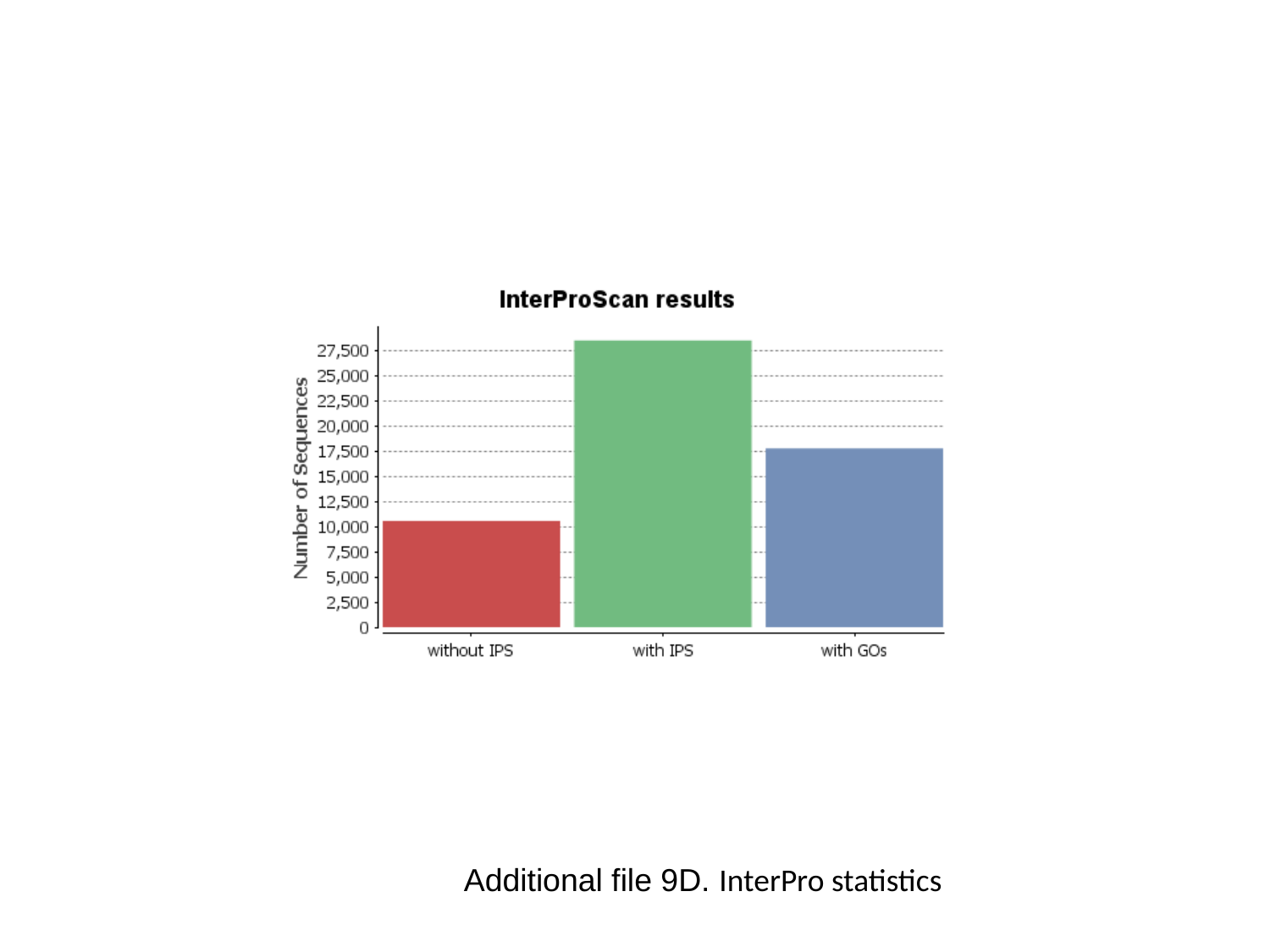

Additional file 9D. InterPro statistics
